# The Inflammatory Response After Ischemic Stroke: Targeting β_2_ and β_1_ Integrins

**DOI:** 10.3389/fnins.2019.00540

**Published:** 2019-05-28

**Authors:** Danielle N. Edwards, Gregory J. Bix

**Affiliations:** ^1^Sanders–Brown Center on Aging, University of Kentucky, Lexington, KY, United States; ^2^Department of Neuroscience, University of Kentucky, Lexington, KY, United States; ^3^Department of Neurology, University of Kentucky, Lexington, KY, United States; ^4^Department of Neurosurgery, University of Kentucky, Lexington, KY, United States

**Keywords:** ischemic stroke, integrins, inflammation, leukocytes, clinical trial results

## Abstract

Ischemic stroke is a leading cause of death and disability with limited therapeutic options. Resulting inflammatory mechanisms after reperfusion (removal of the thrombus) result in cytokine activation, calcium influx, and leukocytic infiltration to the area of ischemia. In particular, leukocytes migrate toward areas of inflammation by use of integrins, particularly integrins β_1_ and β_2_. Integrins have been shown to be necessary for leukocyte adhesion and migration, and thus are of immediate interest in many inflammatory diseases, including ischemic stroke. In this review, we identify the main integrins involved in leukocytic migration following stroke (α_*L*_β_2_, α_D_β_2_, α_4_β_1_, and α_5_β_1_) and targeted clinical therapeutic interventions.

## Introduction

Ischemic stroke is a leading cause of death and disability in the United States with limited therapeutic interventions available, including tissue plasminogen activator (t-PA) and endovascular mechanical thrombectomy (Rao et al., 2014; Benjamin et al., 2017; Rai et al., 2017). These interventions are focused on the removal of the thrombus, restoring blood flow, oxygen and glucose to hypoperfused areas, but are unable to affect the inflammatory, necrotic, and blood-brain barrier (BBB) mechanism that follow. In particular, the initial inflammatory cascade is initiated by the decrease in ATP production, release of cytokines, influx of intracellular calcium, reactive oxygen species, etc., that develops during occlusion and continues for days afterward (Sandoval and Witt, 2008). Using shear forces from cerebral blood flow, marrow-derived leukocytes (including polymorphonuclear leukocytes (PMNs), neutrophils, lymphocytes and monocytes) are recruited to the site of injury (Dereski et al., 1993; del Zoppo, 1994; Stefanidakis and Koivunen, 2006).

For the purpose of this review, we will focus on the recruitment and rolling of leukocytes under the direction of integrins, as well as some of their ligands following reperfusion after ischemic stroke. We will then introduce recent β_2_ and β_1_ integrin-specific stroke clinical trials, and, finally, discuss potential future directions for the field.

## Leukocytic Infiltration Following Ischemic Stroke

### Leukocyte Recruitment

**FIGURE 1:**
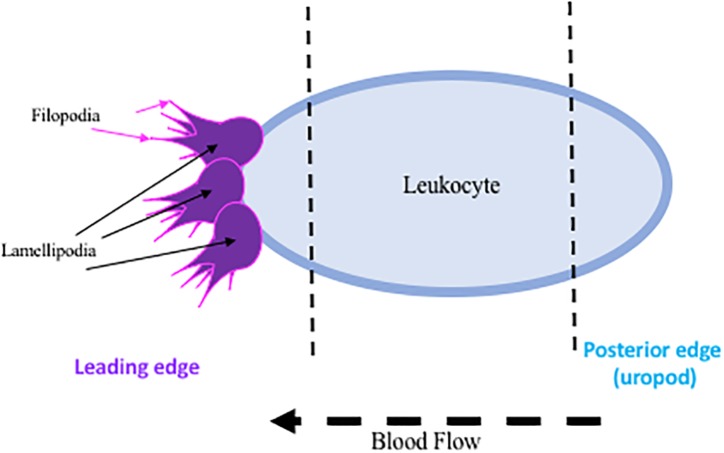
Representative diagram of regions of interest on activated leukocytes.

The initial endothelial response upregulates endothelial selectins, particularly P-selectin and E-selectin, translocating them from an intracellular, inactive state, to the available extracellular matrix for leukocytic binding, while the upregulation of L-selectin on the leukocyte is essential for recruitment to the site of injury (Bargatze et al., 1994). Both are acutely regulated, P-selectin at 15 min and E-selectin at 2 h post ischemia. These extracellularly located selectins then facilitate the recruitment and activation of leukocytes to the area of ischemia (Zhang et al., 1998). Leukocytes then undergo a conformational change, facilitating polarization and the development of certain cellular characteristics: a leading edge, main body, and rear-uropod protrusion. The uropod, or posterior protrusion, in fast moving leukocytes promotes mobility, while the leading protrusions (lamellipodia and filopodia, small leg-like projections) are less likely to be used due to the rate limiting interaction with actin filaments (Ridley et al., 2003) (summarized in Figure 1). Though this mechanism is less obvious in the highly mobile leukocytes. Once leukocytes are bound to selectins, additional binding to integrins and adhesion molecules (intracellular adhesion molecule (ICAMs and vascular adhesion molecule-1 (VCAM) occurs, permitting leukocytic rolling (del Pozo et al., 1995; Lorant et al., 1995; Kindzelskii et al., 1996; Becker, 2002). Additional damage can occur once at the site of ischemia, as infiltration into the brain parenchyma across the BBB destroys surrounding vasculature (Clark et al., 1993; Chou et al., 2004), and leukocytes continually release additional factors (reactive oxygen species, cytokines, and proteases) that enhance leukocytic recruitment (Wang et al., 2008).

### Leukocyte Infiltration

At the site of injury, leukocytes continue to increase binding on cerebrovasculature up to 48 h following ischemic stroke, and use transendothelial migration as a method for infiltration from the cerebrovasculature into the surrounding brain parenchyma. Early adhesion, prior to 24 h following reperfusion, is attributed to neutrophils. Within 30 min to a few hours following reperfusion, neutrophils arrive at the site of injury, peaking at maximum expression around 1–3 days, though expression can still be detected 7–15 days later in preclinical stroke models (Weston et al., 2007). This upregulation is also seen in ischemic stroke patients, where neutrophils have been detected beginning at 6 h, with radiolabeled imaging, and continue to be detected up to 72 h (Aspey et al., 1989). The early recruitment and infiltration of neutrophils across the BBB appears to be highly significant in stroke, as high neutrophilic infiltration is associated with damaged cerebrovasculature (Enzmann et al., 2013), while depletion reduces infarct volume and dysfunction in stroke models (Chou et al., 2004).

As reperfusion injury continues, the circulating leukocytes switch from neutrophils to mononucleuar leukocytes (monocytes/lymphocytes) which dominate the adherent culture from 24 h to 7 days post reperfusion (Schroeter et al., 1994; Stevens et al., 2002; Ishikawa et al., 2005). Of the two types of lymphocytes, B- and T-, T cells have emerged as the dominant, damage-inducing lymphocyte in ischemic stroke (Brait et al., 2011). Preclinical studies have shown that the inhibition of all lymphocytes results in smaller infarct and improved neurological outcomes, but only the reintroduction of T-lymphocytes to mice reversed any benefits (Yilmaz et al., 2006; Kleinschnitz et al., 2010). Activated T-lymphocytes, not B-, have been detected in patients up to 60 days post-stroke, and are correlated with an increased risk of stroke reoccurrence and death (Tarkowski et al., 1995; Nadareishvili et al., 2004).

### Clinical Importance of Leukocytes

Multiple studies have established the importance of leukocytic adherence and infiltration into the brain parenchyma following ischemic stroke, but targeting the leukocytes has a high degree of risk. This is evident as inhibition of leukocytic cells increase the occurrence of bacterial infection and mortality as evident by Leukocytic Adhesion Deficiency (LAD-1) (Stefanidakis and Koivunen, 2006). Because these cells are necessary for bacterial resistance, systemic inhibition following ischemic stroke is exceptionally risky. However, studies in stroke patients show a strong correlation between increased levels of peripheral leukocytes and neutrophils, and increased infarct volume (Price et al., 2004; Buck et al., 2008). Thus, some current therapeutic strategies have focused on the inflammatory cascade have focused on blocking the adhesion and infiltration of cells at the site of injury, primarily endothelial expressed ICAMs and VCAM. This method has shown success in preclinical studies, but has failed to translate to the clinic. Thus, the focus has switched to directly targeting the integrins, a primary mediator of leukocyte adhesion, rather than their ligands, as discussed above. This review is focused on integrins β_2_ and β_1_ that have shown promise in therapeutically targeting the ischemic stroke inflammatory cascade.

## Role of Integrins Post-Stroke: an Overview

Integrins are a diverse group of heterodimers composed of 18 different α and β subunits, creating 24 unique combinations. Integrins exist on every cell type, while exhibiting a high diversity of ligands and grouped into four different receptor groups: RGD (Arg-Gly-Asp), laminin receptors, collagen receptors, and leukocyte-specific receptors. Within these groups, integrins can have a variety of ligands and roles following ischemic stroke (reviewed in Edwards and Bix, 2019). Under normal cerebrovascular conditions, integrins are in a highly inactive state, typically in a bent conformation (Takagi et al., 2002; Nishida et al., 2006). Following ischemic stroke, activation signals are sent. Chemokines are translocated to the lumen, on the apical side of endothelial cells, to induce “inside-out” signaling (Chavakis, 2012). Integrins then undergo a conformational change to increase integrin affinity for potential ligands while enhancing detection by localizing to the leading or rear-facing edge of the leukocyte’s cell wall for ligand detection (Ridley et al., 2003; Hyun et al., 2009). Activated integrins then bind to available ligands, permitting leukocytic rolling and intracellular signaling. This is termed “outside-in” signaling (Hato et al., 1998; Tominaga et al., 1998; Ley et al., 2007). Leukocytes continue movement to the site of injury, looking for areas to cross the endothelial cell barrier, and eventually coming to a halt. Aggregation/clustering of integrins increases binding avidity (strength of binding), preventing flow conditions from detaching leukocytes from the endothelial cells (Ley et al., 2007). Using transmigration, leukocytes will infiltrate into the cerebral parenchyma using these integrin-ligand connections.

### β_2_ Integrins

**FIGURE 2:**
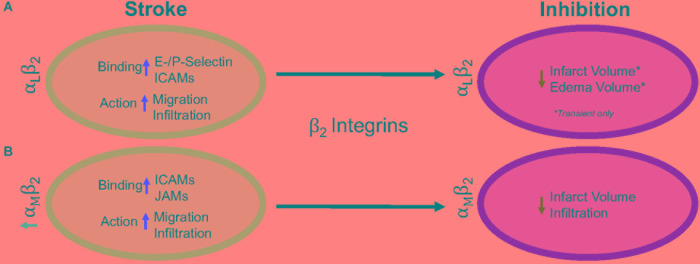
Representative image of the β_2_ integrin response following experimental stroke and inhibitory antibody treatment in preclinical trials. Inhibition of **(A)** α_*L*_β_2_ and **(B)** α_*M*_β_2_ integrins post-stroke responses and effects.

β_2_ integrins are the only group of integrins exclusively expressed on leukocytes (derived from hematopoietic cells) (Schenkel et al., 2004), and like most integrins, are highly conserved across species (Schittenhelm et al., 2017). They are also the most highly expressed integrin on circulating blood leukocytes, tending to cluster at the retraction area of the cell (the rear), in both an active and inactive state, compared to other β_1_, β_4_, β_3_, and β_7_ integrins found on circulating leukocytes (Pierini et al., 2000; Lindbom and Werr, 2002). Genetic leukocyte adhesion changes (LAD-1, as discussed above) has been attributed to mutations in the β_2_ subunit, reducing β_2_ expression. Thus, leukocytic movement is reduced on the cell surface with less movement toward the site of inflammation (Arnaout, 1990; Scharffetter-Kochanek et al., 1998). Importantly, in β_2_ inhibited mice, there is not total arrest of leukocytic recruitment or infiltration (Pierini et al., 2000), suggesting that other factors likely play a role. There are 4 identified heterodimers of β_2_ integrins, and of these, the most highly studied are α_*L*_β_2_ and α_*M*_β_2_ in ischemic stroke, and will be reviewed in more detail below. The other β_2_ integrins, α_*X*_β_2_ and α_D_β_2_, have not been individually studied in the context of stroke as have α_*L*_β_2_ and α_*M*_β_2_ integrins, though CD18 (β_2_) inhibition in addition to t-PA has been shown to increase the time window of t-PA administration without an increase in hemorrhagic transformation in a rat embolic stroke model (Zhang et al., 1999). Furthermore, Figure 2 summarizes the results in this section.

#### α_*L*_β_2_ Integrin

Integrin α_*L*_β_2_ is also referred to as CD11a/CD18 and LFA-1 (lymphocyte functional-associating antigen-1). α_*L*_β2 integrin acutely increases in ischemic stroke patients, with detectable amounts through 72 h associated around the area of ischemia (Gerhard et al., 2000; Zhao et al., 2002). This suggests a correlation between α_*L*_β_2_ integrin expression and inflammatory damage following ischemia. α_*L*_β_2_ is expressed on all leukocytes (Soriano et al., 1999), though at particularly high levels on T-lymphocytes (Hammond et al., 2014; Walling and Kim, 2018). In healthy individuals, extracted blood analysis revealed that α_*L*_β_2_ activation requires leukocytic rolling on P- or E-selectins, inducing an active conformational change (Kuwano et al., 2010), but it is the binding of chemokines g-protein coupled receptors (GPCR) and Rap-1 activation that induces the high-affinity conformational state of α_*L*_β_2_ (Steffen et al., 1994; Greenwood et al., 1995; Ghandour et al., 2007). In this state, α_*L*_β_2_ has many possible ligands, ICAM-1, ICAM-2, ICAM-3, ICAM-4, ICAM-5, and junctional adhesion molecule-1 (JAM-1) (Marlin and Springer, 1987; de Fougerolles et al., 1991, 1994; Tian et al., 2000), though ICAM-1 is preferentially bound (Walling and Kim, 2018). The high avidity α_*L*_β_2_-ICAM-1 complex, once formed, allows t-lymphocytes to move against circulatory flow and the shear forces, resulting in the high-speed movement of leukocytes (Katakai et al., 2013; Dominguez et al., 2015).

In an intraluminal model of experimental ischemic stroke, α_*L*_β_2_ inhibition with the use of transgenic mice results in reduced infarct volume, edema volume and mortality. However, this phenomenon is evident in transient, but not permanent middle cerebral artery occlusion (Arumugam et al., 2004). This may be due to the previously mentioned high avidity of α_*L*_β_2_-ICAM-1 bonds, and is evident in an *in vitro* study using α_*L*_β_2_ (LFA-1) knock-in mice that experience high avidity through binding of lymphocytes mediated through ICAM-1 binding, but are unable to continue movement due to a non-polarized uropod (Park et al., 2010). An explanation for this phenomenon may be that the recycling process within the leukocyte is overwhelmed (Shaw et al., 2004). By enhancing α_*L*_β_2_ expression, recycling may not be able to allow for dislocation of α_*L*_β_2_-ICAM-1 complexes, preventing movement from the loss of high adhesion bonds. Enhanced α_*L*_β_2_ expression could be a potential new avenue for therapy, especially if no enhanced mortality, infection, etc., are observed.

Independently, ICAMs play a significant role in inflammation following ischemic stroke. ICAM-1, in particular, is acutely increased in both cultured human endothelial cells undergoing hypoxia and following intraluminal suture middle cerebral artery occlusion, while expression remains sustained for up to a week post-injury (Hess et al., 1994a,b; Zhang et al., 1995). ICAM-2, another possible ligand, does not change in expression following cytokine stimulation (de Fougerolles et al., 1991; Nortamo et al., 1991a,b). Furthermore, serum of ischemic stroke patients contains soluble ICAM-1, but not ICAM-2 in addition to being a risk factor (Kaplanski et al., 1994; Shyu et al., 1997). Antibodies targeting ICAM-1 in rodents and humans have shown contradictory results. An intraluminal suture middle cerebral artery occlusion model in mice and rats showed a decrease in leukocyte infiltration and infarct volume (Connolly et al., 1996; Kitagawa et al., 1998; Vemuganti et al., 2004), while one study reported opposing effects (Furuya et al., 2001). ICAM-1 inhibition was translated to the clinic through testing of the murine ICAM-1 antibody, Enlimomab in ischemic stroke. Unfortunately, the study was halted early due to increased rate of infection, infarct volumes, neurological scores and mortality for patients (Furuya et al., 2001).

#### α_*M*_β_2_ Integrin

Integrin α_*M*_β_2_, also known as CD11b/CD18 and Mac-1 (macrophage-1 antigen)_,_ exhibits many similarities to α_*L*_β_2_ through its expression on all leukocytes (Springer et al., 1979), and common ligand binding partners such as the family of ICAMs and JAMs (von Andrian et al., 1991). Additional ligands are fibrinogen, heparin (von Andrian et al., 1991), elastase (Cai and Wright, 1996), complement C3 fragment (C3bi) (Micklem and Sim, 1985), kinogen components, and urokinase and its receptor (Chavakis et al., 1999). Just as α_*L*_β_2_, hypoxia induced factors (cytokines, chemokines, etc.) induce conformational change of α_*M*_β_2_ to a high affinity ligand-binding state (Stanimirovic et al., 1997). Binding assays with ICAM-1 as a ligand and both α_*L*_β_2_ and α_*M*_β_2_ as receptors show α_*L*_β_2_ integrin is preferably bound (Lub et al., 1996). This suggests that the binding sites on both α_*L*_β_2_ and α_*M*_β_2_ compete for ICAM-1 binding.

Following experimental ischemic stroke in rats, integrin α_*M*_β_2_ is upregulated (Campanella et al., 2002), and has shown benefit when inhibited. Antibodies against both CD11b/CD18 reduce infarct volume and reestablish cerebral blood flow as a result of decreased neutrophil infiltration following intraluminal stroke surgery (Chen et al., 1994; Bowes et al., 1995; Zhang et al., 1995). In a different approach, the addition of recombinant neutrophil inhibitory factor (rNIF) inhibits a binding domain on Mac-1 and yields similar results in the same intraluminal occlusion model (Jiang et al., 1998). Furthermore, and similarly to α_*L*_β_2_ integrin inhibition, inhibition of α_*M*_β_2_ is also effective in transient, but not permanent experimental ischemic stroke in an embolic occlusion model (Zhang et al., 2003).

### β_1_ Integrins

**FIGURE 3:**
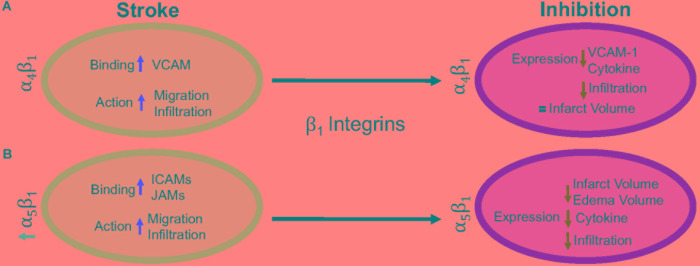
Representative image of the β_1_ integrin response following experimental stroke and inhibitory antibody treatment in preclinical trials. Inhibition of **(A)** α_4_β_1_ and **(B)** α_5_β_1_ integrins post-stroke responses and effects.

β_1_ integrins are a diverse set of integrins, with laminin-binding, collagen-binding, RGD-binding and leukocyte heterodimers. β_1_ integrins are not as highly expressed on leukocytes as β_2_ integrins, but they do play a major role in leukocyte adhesion and migration following ischemic stroke. The activity of β_1_ integrins is similar to β_2_ integrins. They undergo a conformational change to induce “inside-out” and “outside-in” cellular signaling (Campanero et al., 1994). As the cells migrate, the β_1_ integrins are most commonly clustered around the uropod, but will be located in any area of the leukocyte that is in contact with the endothelial cell or extracellular matrix (Campanero et al., 1994; Caimi et al., 2001). Inhibition of the β_1_ integrin, just as with β_2_ integrin inhibition, does not fully stop leukocyte rolling. However, when both β_1_ and β_2_ integrins are inhibited, complete leukocyte arrest occurs (Lobb and Hemler, 1994; Pierini et al., 2000). This suggests that both β_1_ and β_2_ integrins are necessary for leukocyte migration, regardless of expression load. Of all the β_1_ integrins, both α_4_β_1_ and α_5_β_1_ appear to be the most highly expressed and the most studied in post-stroke inflammation. The other β_1_ integrin expressed on leukocytes, α_9_β_1_, has not been studied in the context of stroke as its expression and role has not yet been fully elucidated in the brain. Figure 3 summarizes the results discussed in this section.

#### α_4_β_1_ Integrin

α_4_β_1_, also known as CD49d/CD29 VLA-4 (very late antigen-4)_,_ is localized primarily to leukocytes (neutrophils, monocytes, lymphocytes, macrophages, etc.) and microglia as a leukocyte-specific receptor. Additionally, α_4_ will also dimerize with β_4_, which is found in gut endothelium (Hammond et al., 2014). Activation of α_4_β_1_ integrin results from the binding of upregulated chemokines to GPCRs in the same manner as α_*L*_β_2_ as discussed above (Vajkoczy et al., 2001). This stimulates binding to α_4_β_1_’s preferred ligand, VCAM-1, but experiments have shown some preference for paxillin ICAM-1 (Steffen et al., 1994; Ghandour et al., 2007), and fibronectin (Hart and Greaves, 2010) as well. Interestingly, instead of using the β_1_ submit of the heterodimer for binding, integrin α_4_β_1_ uses its α subunit of α_4_β_1_ to mediate binding to VCAM-1 (Luo et al., 2007).

Preclinical ischemic stroke studies targeting α_4_β_1_ have shown increasingly varied results. Most researchers reported a decrease in VCAM-1 expression, cytokine production, and infiltrating leukocytes (Liesz et al., 2011; Langhauser et al., 2014; Llovera et al., 2015), but this reduction in inflammation did not result in reduced infarct volumes or functional deficit following analysis of a randomized preclinical trial involving six different centers (Llovera et al., 2015). Langhauser et al went one step further and found that no treatment paradigm (prophylactic or therapeutic) and no model (transient or permanent) showed efficacy (Langhauser et al., 2014). On the other hand, both Becker, 2002 and Relton et al., 2000 found that inhibition of α_4_ improved both infarct volumes and functional deficits. When a preclinical randomized control trial was implemented at multiple centers, researchers found efficacy only in patients with small infarct volumes (Llovera et al., 2015). Collectively, these contradictory results may be caused by a couple of scenarios, 1) the varying expression of integrin α_4_β_1_ expression following ischemic stroke resulting in continued leukocyte infiltration, or 2) integrin α_4_β_1_ is not a primary driver of post-stroke pathophysiology, but other factors, including other integrins, promote leukocyte migration (Hammond et al., 2014).

#### α_5_β_1_ Integrin

α_5_β_1,_ also known as CD49e/CD29 and VLA-5, plays an as yet largely undetermined role in inflammation, with studies primarily limited to cell culture. What is known is that leukocytes express different β_1_ integrins with α_5_β_1_ composing around 50% of all β_1_ –integrins expressed on neutrophils (Pierini et al., 2000) and monocytes (Pacifici et al., 1994). Additionally, α_5_β_1_ is necessary for leukocyte adhesion. Only inhibition of both α_5_β_1_ and β_2_ integrins completely blocks adhesion *in vitro* (Pierini et al., 2000), while inhibition of α_5_β_1_ alone prevents transmigration across the BBB (Labus et al., 2018) *in vitro*. As an RGD receptor, fibronectin has been shown to be the primary and preferred [over other potential ligands such as fibrinogen (Suehiro et al., 1997)] ligand for α_5_β_1_ on endothelial cells and leukocytes (Schaffner et al., 2013; Bharadwaj et al., 2017). Importantly, in the presence of activated α_L_β_2,_ leukocyte α_5_β_1_ binding to fibronectin is enhanced (Bohnsack, 1992; Loike et al., 1999; Gronholm et al., 2016). α_5_β_1_ integrin expression is induced by cytokines, particularly TNFα (Li et al., 2011) toward the leading edge of the cell in contrast with other integrins at the uropod (Pierini et al., 2000). Furthermore, α_5_β_1_ integrin appears to be highly sensitive to calcium (Pierini et al., 2000), an ion that is increased rapidly following reperfusion (Sandoval and Witt, 2008). Upon calcium buffering, α_5_β_1_ expression moves from the front of the cell to the uropod and the leukocyte becomes elongated. The change in expression localization and morphology is attributed to non-movement as the leukocyte cannot detach α_5_β_1_ from the vascular wall (Pierini et al., 2000). Recently, Edwards et al. (2019) found that inhibition of α_5_β_1_ integrin by the small peptide ATN-161 prevented CD45+ leukocytes from infiltrating the brain parenchyma following the tandem/transient common carotid artery/middle cerebral artery occlusion model. Additionally, mice were observed to have reduced BBB permeability, functional deficits, edema, and infarct volume following middle cerebral artery occlusion (Roberts et al., 2015; Edwards et al., 2019). Thus, targeting α_5_β_1_ after ischemic stroke could be a new avenue for reduction of inflammation following ischemic stroke.

## Clinical Implications

The preclinical studies discussed here point towards the potential of targeting β_2_ and β_1_ integrins in the treatment of post-stroke inflammation. Though their potential has not fully been elucidated, many clinical trials, not just limited to stroke, have been approved in the last 10 years targeting these integrins.

The most common target for post-stroke inflammation are the β_2_ integrins. Though some efficacy has been reported, no clinical stroke trials to date have targeted the α_L_ subunit in stroke patients. However, one clinical trial with the monoclonal antibody, Efalizumab, has shown promise in decreasing T-lymphocyte rolling in patients with moderate-severe plaque psoriasis (Lebwhohl et al., 2003).

In preclinical studies targeting α_*M*_β_2_, a hookworm isolated recombinant glycoprotein targeting rNIF (UK279276) (Zhang et al., 2003) and humanized Hu23F2G (Leukarrest) (Yenari et al., 1998), were both shown to decrease infarct volume and increase functional recovery following reperfusion. Both therapies had negligible side effects in Phase 1 studies and thus were continued to a Phase II study, respectively, before the trials were halted due to no observed efficacy (Becker, 2002; Krams et al., 2003). The failure to target α_*M*_β_2_ integrin may be due to the observation that human ischemic stroke patients do not experience the increase in α_*M*_β_2_ expression as seen in rodent stroke models (Caimi et al., 2001). Interestingly, when given in conjunction with United Kingdom279276, patients experienced a slight improvement (Krams et al., 2003), but no follow-up has been conducted. This interesting effect may be worth additional investigation in future clinical trials.

Clinical inhibition of β_1_ integrins, on the other hand, is small and varied. Of the current clinical trials, one trial has emerged targeting α_4_β_1_ in the context of ischemic stroke. The monoclonal antibody targeting the α_4_ subunit (Natalizumab) has been successful in protecting patients from relapses in multiple sclerosis (Polman et al., 2006) and Crohn’s disease (Sandborn et al., 2015). However, in a Phase II ischemic stroke study, patients receiving Natalizumab showed no improvement in infarct growth or neurological scores over 30 days. Furthermore, two patients (out of 79) died from serious infections attributed to Natalizumab treatment (Elkins et al., 2017). At this time, there are no further clinical trials planned.

## Future Considerations

As discussed in this review, targeting leukocytic integrins has had limited to no efficacy in clinical trials. Importantly, these studies have collectively employed only three different therapeutics and two targets; there are still significant areas that can be investigated. Though not discussed here, most preclinical investigations have focused on the ligands themselves rather than the integrin as the therapeutic target, highlighting the continued importance of integrins in stroke.

It is also important to note that preclinical studies carried out in rodents inadequately model the post-stroke pathophysiology that patients experience. Preclinical stroke research is also typically limited, focusing on one species, sex, and age that do not necessarily match the demographic of stroke patients (see Kahle and Bix, 2012 for a review of this topic). Furthermore, as the changes following stroke and/or reperfusion are inadequately understood, identifying appropriate therapeutic targets that translate from the lab to clinical trials, has been particularly challenging.

However, this does not suggest abandoning therapeutic trials for ischemic stroke. As mentioned above, stroke is a leading cause of death and disability, separate from cardiovascular disease. This will not improve without intervention with our aging and obese population. Fortunately, with the advent of stroke mortality-altering therapies, i.e., t-PA and endovascular mechanical thrombectomy, our financial burden has shifted to aftercare. When we review the amount of trials performed for thrombolytic agents (Multicentre Acute Stroke Trial–Italy (MAST-I) Group, 1995; National Institute of Neurological Disorders and Stroke rt-Pa Stroke Study Group, 1995; The Multicenter Acute Stroke Trial–Europe Study Group, 1996) and endovascular thrombectomy [MR CLEAN (Berkhemer et al., 2015), ESCAPE (Goyal et al., 2015), EXTEND IA (Campbell et al., 2015), SWIFT PRIME (Saver et al., 2015), and REVASCAT (Jovin et al., 2015)] as potential treatments of ischemic stroke, it is obvious that the complexities of stroke affect the outcome of the clinical trial. This includes, but is not limited to, the time a patient takes to arrive at an ER, time to treatment, location of the stroke, amount of surrounding collaterals, current medications and co-morbidities (diabetes, cancer, etc.), and if the patient has experienced multiple strokes.

Based on current advances, as well as previous failures, a focus on integrins as a therapeutic target for stroke is emerging. A significant reason for this focus may be the complex, multi-dimensional role that integrins appear to play in brain pathophysiology. Integrins are diverse, existing on all cell types with varying roles depending upon expression and activation. This complexity can represent a significant challenge to integrin-targeted therapies inasmuch as such therapies could have diverse, even unintended off-target effects. However, we believe that this can be overcome by a better understanding of how integrin function and expression is altered after stroke, with the potential to exploit stroke-dependent integrin changes to therapeutic effect. For example, identifying a specific integrin to be upregulated in select cells in the post-stroke brain or brain-targeting cells, but not in other organs, could render it a viable therapeutic target. This emphasizes the need and importance of preclinical stroke research to discover and unravel the complexities of integrin biology. We are confident that such studies will result in viable new stroke therapies.

## Conclusion

In this review, we have implicated integrins as an area of research for limiting inflammation following ischemic stroke. To date, therapeutic inhibition of α_*L*_β_2_, α_*M*_β_2_, and α_4_β_1_ has shown promising results in preclinical studies, but translation to the clinic has been disappointing. Going forward, more targeted antibodies to all reactive β_1_ and β_2_ integrins after ischemic stroke may prove more beneficial, but more research needs to be done to completely understand the human inflammatory response and how that relates to changes in preclinical models.

## Author Contributions

DE wrote the manuscript. DE and GB edited the manuscript.

## Conflict of Interest Statement

The authors declare that the research was conducted in the absence of any commercial or financial relationships that could be construed as a potential conflict of interest.
